# Machine learning algorithms using national registry data to predict loss to follow- up during tuberculosis treatment

**DOI:** 10.21203/rs.3.rs-3706875/v1

**Published:** 2023-12-11

**Authors:** Moreno M. S. Rodrigues, Beatriz Barreto-Duarte, Caian L. Vinhaes, Mariana Araújo-Pereira, Eduardo R. Fukutani, Keityane Bone Bergamaschi, Afrânio Kristki, Marcelo Cordeiro-Santos, Valeria C. Rolla, Timothy R. Sterling, Artur T. L. Queiroz, Bruno B. Andrade

**Affiliations:** Fundação Oswaldo Cruz; Universidade Salvador; Escola Bahiana de Medicina e Saúde Pública; Centro Universitário Faculdade de Tecnologia e Ciências; Instituto Gonçalo Moniz, Fundação Oswaldo Cruz; Fundação Oswaldo Cruz; Universidade Federal do Rio de Janeiro; Fundação Medicina Tropical Doutor Heitor Vieira Dourado; Instituto Nacional de Infectologia Evandro Chagas; Vanderbilt University School of Medicine; Fundação Oswaldo Cruz; Instituto Gonçalo Moniz, Fundação Oswaldo Cruz

**Keywords:** tuberculosis, score prediction, loss to follow-up, machine learning

## Abstract

**Background:**

Identifying patients at increased risk of loss to follow-up (LTFU) is key to developing strategies to optimize the clinical management of tuberculosis (TB). The use of national registry data in prediction models may be a useful tool to inform healthcare workers about risk of LTFU. Here we developed a score to predict the risk of LTFU during anti-TB treatment (ATT) in a nationwide cohort of cases using clinical data reported to the Brazilian Notifiable Disease Information System (SINAN).

**Methods:**

We performed a retrospective study of all TB cases reported to SINAN between 2015–2022; excluding children (<18 years-old), vulnerable groups or drug-resistant TB. For the score, data before treatment initiation were used. We trained and internally validated three different prediction scoring systems, based on Logistic Regression, Random Forest, and Light Gradient Boosting. Before applying our models we split our data into train (~80% data) and test (~20%), and then we compare model metrics using a test data set.

**Results:**

Of the 243,726 cases included, 41,373 experienced LTFU whereas 202,353 were successfully treated and cured. The groups were different with regards to several clinical and sociodemographic characteristics. The directly observed treatment (DOT) was unbalanced between the groups with lower prevalence in those who were LTFU. Three models were developed to predict LTFU using 8 features (prior TB, drug use, age, sex, HIV infection and schooling level) with different score composition approaches. Those prediction scoring system exhibited an area under the curve (AUC) ranging between 0.71 and 0.72. The Light Gradient Boosting technique resulted in the best prediction performance, weighting specificity, and sensibility. A user-friendly web calculator app was created (https://tbprediction.herokuapp.com/) to facilitate implementation.

**Conclusions:**

Our nationwide risk score predicts the risk of LTFU during ATT in Brazilian adults prior to treatment commencement. This is a potential tool to assist in decision-making strategies to guide resource allocation, DOT indications, and improve TB treatment adherence.

## INTRODUCTION

Despite the widespread availability of curative treatment of tuberculosis (TB), this disease remains a major plague of humanity, accounting for more than one million deaths annually[1]. Global treatment success is still below the targets established by the World Health Organization (WHO)[2, 3], especially in low- and middle-income countries (LMIC) such as Brazil[4].

Current WHO treatment recommendations for drug-susceptible TB include six months of a combination of antibiotics[1]. Such long treatment is associated with an increased risk of LTFU and may lead to adverse drug reactions[2]. Early identification of patients at high risk of LTFU at the moment of diagnosis with clinical and sociodemographic characteristics is key to providing personalized care, which may involve directly observed treatment (DOT), and helping decision-making strategies to mitigate losses in the cascade of care. To do so, the establishment of reliable and accurate prediction tools[4] is necessary, especially in low-resource settings with a high-middle TB disease burden.

Brazil is among the countries with the highest number of TB cases in the world, despite the fact that it follows the WHO’s standardized TB treatment recommendations. Importantly, the cascade of treatment care in Brazil is composed of 3 steps: 1) mandatory reporting of TB cases to the Notifiable Diseases Information System (SINAN)[5, 6]; 2) a six-month treatment regimen, usually in fixed-dose combination (FDC)[7]; and 3) treatment-associated outcomes are reported in the SINAN database. Thus, this is a significant source of data that could be explored to develop prediction models for LTFU during ATT.

Therefore, we developed a prediction model for LTFU among pulmonary TB treatment cases in Brazil. We used publicly available data from SINAN-TB and applied machine-learning methods to choose the most accurate technique. We aimed to test the ability of models to predict a LTFU at the baseline consultation utilizing only data clinical and sociodemographic data that is easily obtained at diagnosis. Importantly, the developed a model that could be used by both the Brazilian government and clinicians as a readily available web-based tool for decision-making to achieve higher rates of TB treatment success.

## MATERIALS AND METHODS

### Ethics Statement

All data accessed in this study were obtained from a publicly available platform and pre-processed by the Brazilian Ministry of Health (https://datasus.saude.gov.br) This processing verified the data regarding consistency, duplicate registration, and completeness, following the instructions set by Resolution Number 466/12 on Research Ethics of the National Health Council, Brazil. There was no identifiable information in the databases and thus the study was exempt from approval by ethics committees.

### Study Population

We performed a retrospective analysis of de-identified data from pulmonary TB cases reported to the Brazilian Notifiable Diseases Information System (SINAN).

SINAN is a centralized system for the notification of transmissible diseases, including TB. Data stored in SINAN are maintained by the Brazilian Ministry of Health specifically by the DATASUS (the Information Technology Department of the Brazilian Unified Health System) and can be accessed through a file transfer protocol[6].

**Figure 1:**
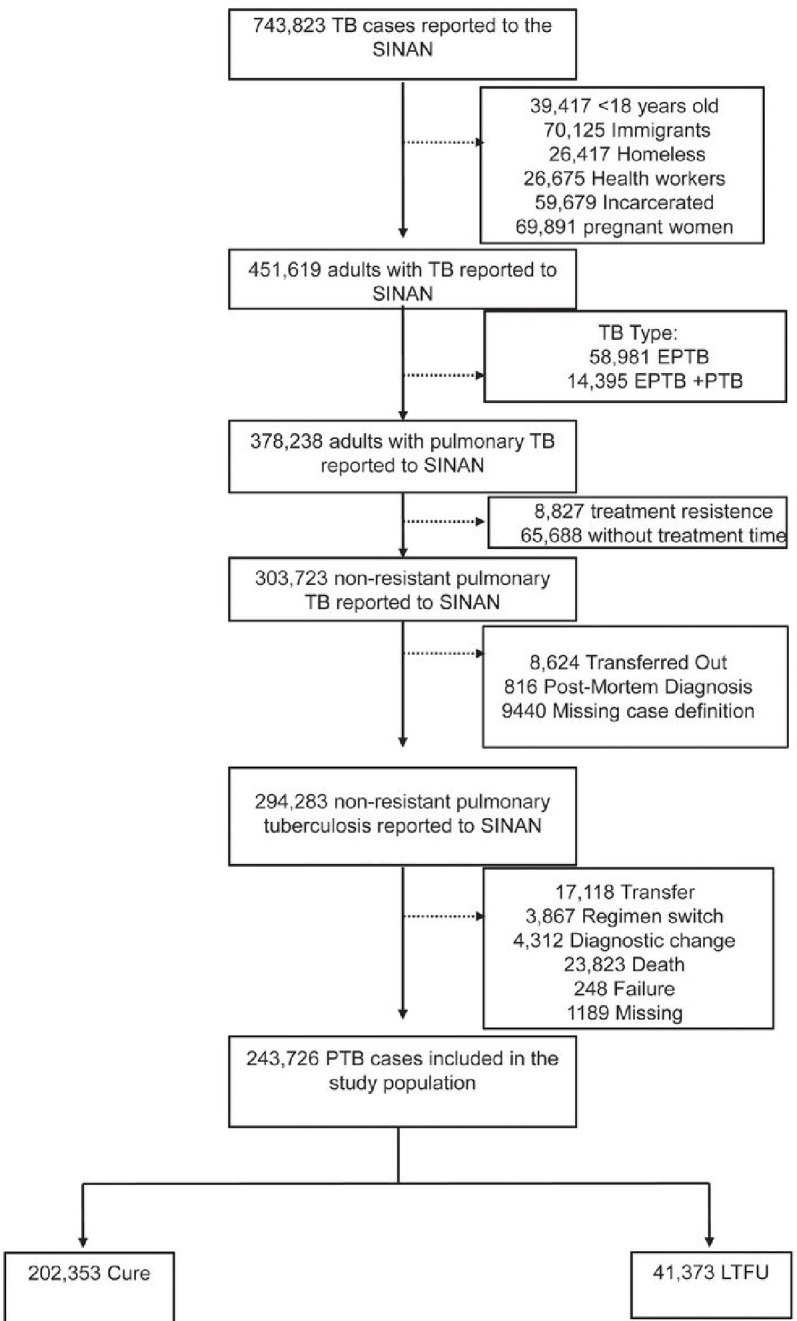
Flowchart demonstrating the study population. Abbreviations: TB: Tuberculosis; LTFU: Loss to follow up; EPTB: extrapulmonary tuberculosis; PTB: pulmonary tuberculosis; HCU: health care unit; .

We included in our study all individuals 18 years old or older, notified in SINAN with pulmonary TB from 2015 through 2022. We exclude from our study any patient that: (i) postmortem TB diagnosed; (ii) belongs to any special population (i.e homelessness, liberty deprivation, pregnant, immigrants, and health worker), (iii) is resistant to any drug (rifampin, isoniazid, pyrazinamide, or ethambutol), and (iv) outcome other than cure or LTFU. (Fig. 1).

### Data analyses

We divided our data analysis process into seven portions/steps: (i) descriptive analyses, (ii) data under sample, (iii) split data, (iv) feature elimination, (v) hyper-parameters tuning, (vi) model evaluation, and (vii) model building. To conduct descriptive analysis we used median followed by interval interquartile (IQR) to describe continuous variable and absolute and relative frequency to categorical. As our data could be considered imbalanced (i.e. ~3 cures for 1 LTFU) we performed an under sample of the most frequent class. Hence, the data set resulting from this process has the same proportion of outcome (i.e. 1 cure for 1 LTFU), and then we split in train test data. The training set was composed by 70% of the total data whereas 30% was kept for model evaluation. To reduce data dimensionality, we used Recursive Feature Elimination using Cross-Validation (RFECV). In this case, we selected RF as the estimator and used it in a 10-fold stratified cross-validation, then we selected the minimum number of variables that leads to the higher model accuracy following the elbow rule. To find the best set of parameters we used the grid search approach, thus for each model (i.e. Logistic Regression, Random Forest, and Light Gradient Boosting) we created a grid of parameters, in the train set we evaluated the best combination of the parameters. To select the best algorithm evaluation, we applied each model with its best combination of parameters to the test set. We then evaluate AUC, accuracy, sensitivity, and specificity. To understand the feature importance and feature contribution to each outcome on a global and local level we used Shapley values. The last step consisted of retraining the model using the whole data set[8–19].

## RESULTS

### Characteristics of the Overall Study population

Between, 2015, and 2022, 743,823 TB cases were notified in SINAN. 243,726 were included in the final study population, with 500,097 (~ 67%) of notifications removed according to our exclusion criteria (Fig. 1). The selected population was stratified according to 202,353 cases that experienced cure and 41,373 experienced LTFU (Fig. 1). At the time of the TB diagnosis, the LTFU group was younger (median age _LTFU_: 37.1 vs. _Cure_ 42.1 years), had more self-identified as non-white (_LTFU_ 72.8% vs. _Cure_65.3%), with lower schooling rates (≥ 12 years, _LTFU_ 2.33% vs. _Cure_6.38%) highest prevalence of HIV infection (_LTFU_ 13.2% vs. _Cure_5.99%) and prior TB (_LTFU_32.4% vs. _Cure_ 10.8%). Among consumption habits, the LTFU group presented the highest prevalence of all the consumption habits evaluated, such as alcohol use (_LTFU_ 29.0% vs. _Cure_16.1%), tobacco use (_LTFU_ 35.0% vs. _Cure_21.9%) and drug use (_LTFU_ 28.6% vs. _Cure_9.12%). Interestingly diabetes was less prevalent in LTFU group (_LTFU_ 5.67% vs. _Cure_10.9%). Noteworthy, the DOT was more prevalent among the cure group (_LTFU_ 21.2% vs. _Cure_ 41.4%). All the evaluated characteristics were statistically significant between the groups (Table 1).

### Comparing machine learning algorithms to predict LTFU

**Figure 2:**
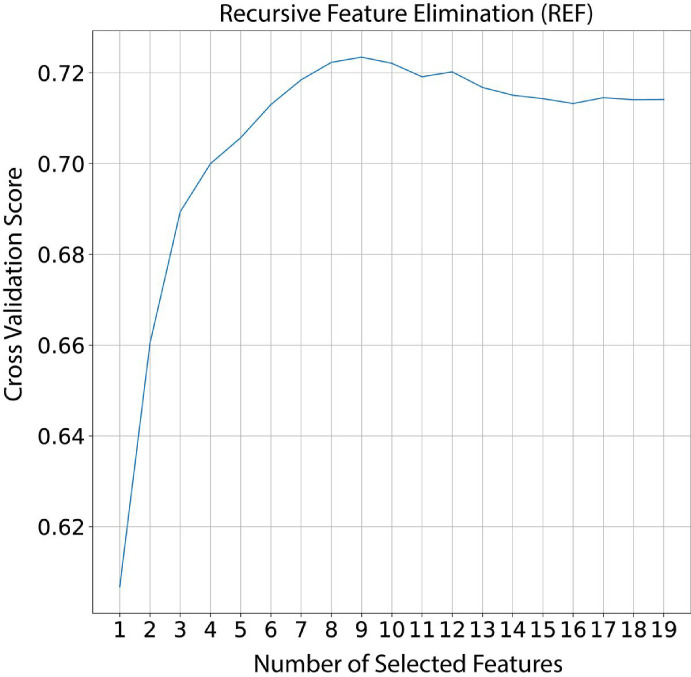
Recursive feature selection elimination. In the x-axis indicating the number of features used by the model while in the y-axis indicating the AUC achieved during the cross-validation

We initiated our model development with 15 variables of which 8 were selected as the most informative by our RFECV approach (Fig. 2): (i) schooling, (ii) sex, (iii) prior TB, (iv) HIV infection, (v) alcohol use, (vi) drug use, (vii) tobacco use and (vii) age. To predict those patients who are more likely to experience an LTFU we proposed three different models using the variables listed above. In our investigation into predicting patient outcomes, three diverse models were employed, each revealing unique hyperparameter preferences for optimal performance. The logistic regression model demonstrated its peak predictive capabilities with a strong regularization, notably C = 0.01. This underscored the critical role of regularization strength in striking a balance between model complexity and generalization. The Random Forest model achieved its optimal performance with a max depth of 8 and a total of 500 decision (no. of estimators) in the ensemble, reflecting the importance of these hyperparameter choices in enhancing predictive accuracy. In the case of the Light Gradient Boosting model, optimal performance was achieved with trees of max depth 4, 500 decision trees (no. of estimators), and a learning rate of 0.01. These results highlighted the intricate interplay between tree complexity, ensemble size, and the learning rate in achieving superior predictive capabilities. These findings shed light on the nuanced preferences of each model, providing valuable insights into the specific hyperparameter configurations that optimize predictive accuracy in the intricate landscape of patient outcome prediction. Such tailored considerations are imperative for the effective application of machine learning approaches to healthcare data.

**Figure 3:**
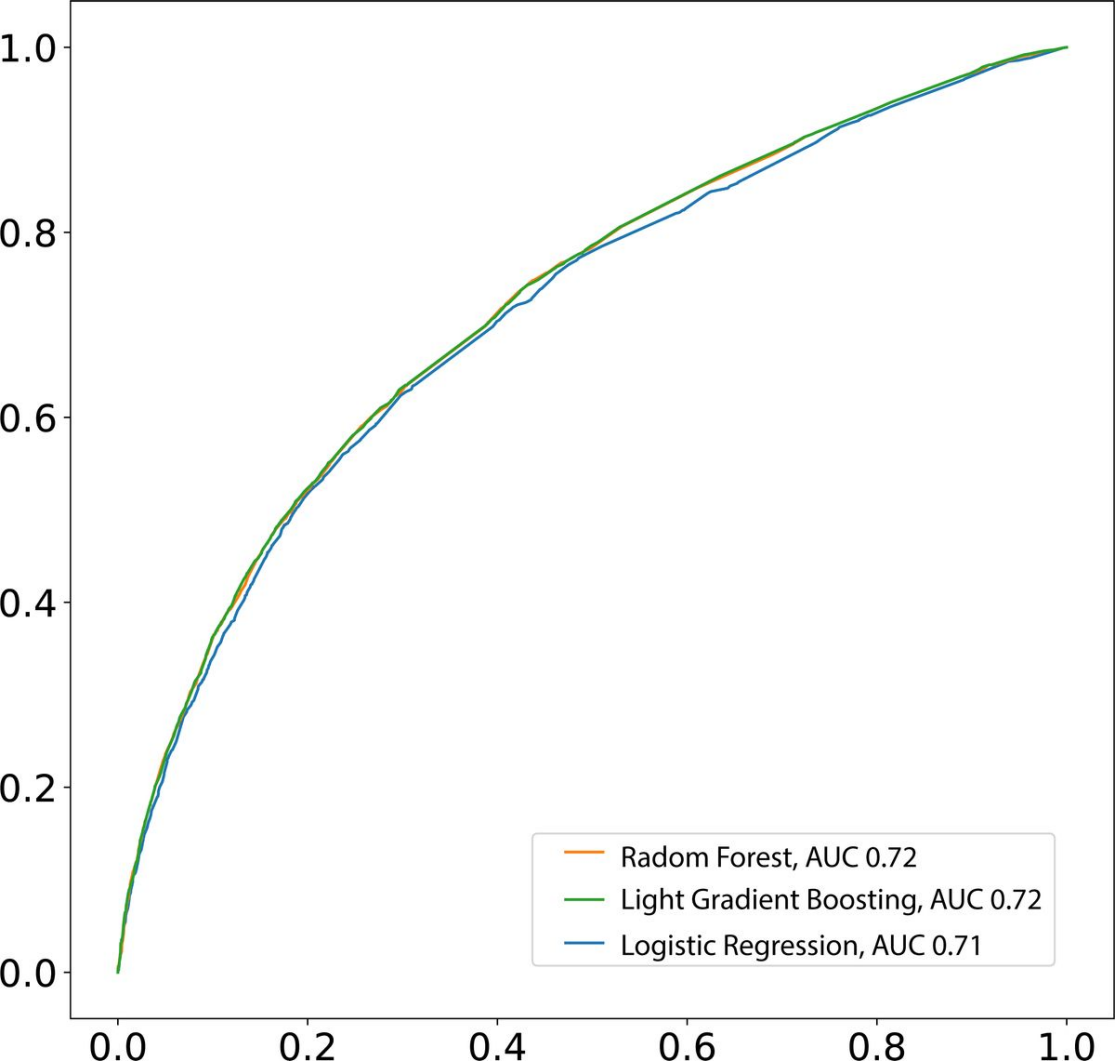
Receiver operating characteristic curve (ROC) for prediction of LTFU based on data available in SINAN ufing three different Machine Learning algorithm.

The next phase consisted of evaluating the three models (using the parameters described above) on the test set. In this case, we found that classifiers presented similar results (Table 2), for example, logistic regression presents an AUC of 0.72 (95% CI = 0.71–0.72) whereas booth Radom Forest and Light Gradient Boosting present an AUC of 0.72 (95% CI = 0.72–0.73) (Fig. 3). When we consider accuracy, we found that all models achieve the same result 0.67 in all the models evaluated (Table 2). However, when we consider sensitivity and specificity models had a different performance. The Logistic Regression model presented the highest specificity (0.75) and the lowest sensitivity (0.58), whereas the Light Gradient Boosting and Radom Forest presented the same sensitivity (0.62) however Light Gradient Boosting presented a higher specificity (0.72) compared to Random Forest (0.70).

**Figure 4:**
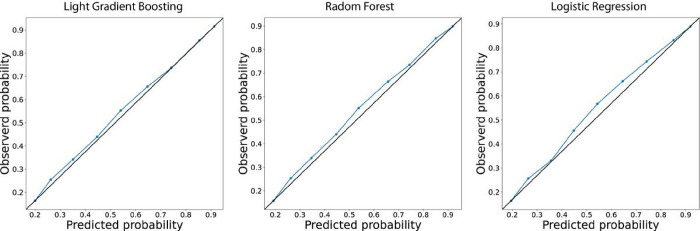
Calibration plot. The x-axis indicating the probability of been predicted as LTFU by the model while the y-axis shows the observed probability. Black line indicates a perfect relation about predicted and observed probability. Blue lines indicate the relation between predicted and observed probability or (a) LightGradient Boosting, (b) Random Forest and (c) Logistic regression

**Figure 5:**
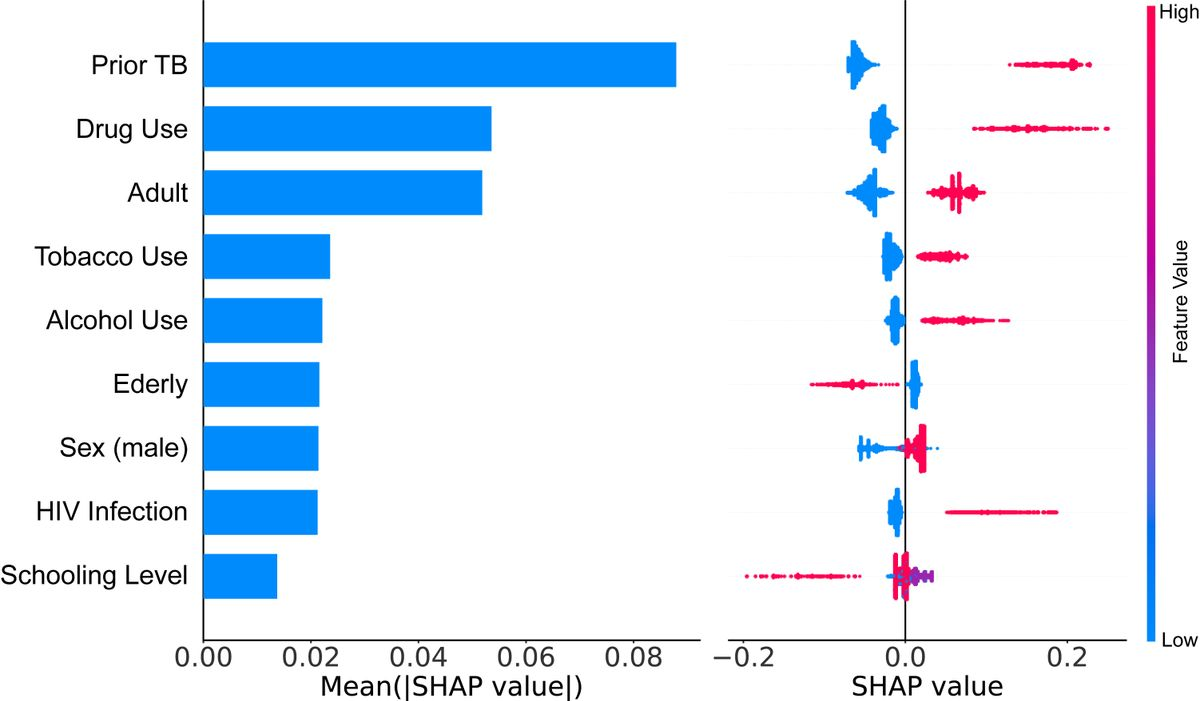
Feature importance computed using SHAP-values on test set. and Relationship between feature value and treatment outcome. Blue dot indicates Cure and, for categorical features the value of no. Red dots indicating LTFU patients and, for categorical features the value of Yes.

According to our calibration plot, the Light Gradient Boosting presented the best result since the predicted probability of an LTFU corresponds to the true likelihood of the positive class being true (Fig. 4). The Random Forest presented the worst result. In this case, the model probability underestimated the real likelihood of the positive class (Fig. 4). Thus based, on all the results we found, we decided to use the Light Gradient Boosting to construct our predictive model. We used SHAP values to allocate the contribution of each feature to a model’s prediction, offering insights into feature importance and interactions. Such values help interpret complex models, providing a nuanced understanding of the factors influencing specific predictions. According to our model, previous TB was the most important feature. In this case, a patient who experienced prior TB had increased likelihood to evolve to LTFU. Another important feature was drug use. Patients who reported to use drugs had the probability of evolve to LTFU during an ATT increased (Fig. 5).

## DISCUSSION

In this study of pulmonary TB cases reported to SINAN in Brazil, we developed a risk score that effectively stratified before treatment initiation those TB cases at higher risk of LTFU during ATT. Our score used data from 8 features, all of which were from the case notification form, and were publicly available. Those features included clinical and epidemiologic information, that can be collected by health professionals before treatment initiation, and which predicted LTFU independent of other characteristics. The use of this risk score could potentially provide crucial information to target specific patients since the diagnosis and improve the successful ATT completion, potentially facilitating the achievement of the WHO target of 90% of patients with treatment success[20].

Importantly, in our study, 14.5% of the total population experienced LTFU, which represents an important problem for public health because of the risk of *M. tuberculosis* transmission; drug-resistant strains can also be generated[21]. Importantly, the rates of DOT in the group that experienced the LTFU were significantly lower than the cure group. Enhancing the importance of the detection of these patients at the beginning of TB treatment might help clinicians in choosing priorities for DOT and the target populations for the Brazilian national TB program.

Our probabilistic score was developed using clinical and sociodemographic data readily collected in most clinical care settings, even in resource-limited settings. Among the variables selected, prior TB, consumption habits (alcohol, tobacco, or drug use), age (adult and elderly), biological sex, HIV infection, and schooling level were the risk factors that most contributed to an LTFU during TB treatment. Some of these characteristics have been explored and linked to unfavorable TB treatment outcomes through the relationship with poor therapy adherence, LTFU, and treatment discontinuation[11, 22–28].

In a previous study, a similar score was developed to predict unfavorable anti-TB treatment outcomes in people living with diabetes from China, however using clinical and radiologic data[24]. Another study from Mexico developed an algorithm to predict mortality, failure, and drug resistance in newly diagnosed TB patients with clinical features and laboratory tests[28]. In contrast, our score could be applied in patients with or without diabetes, by utilizing only clinical information, without the necessity of laboratory data or radiographic exams.

While exploring data from the RePORT-Brazil consortium, we have previously reported a clinical prediction model for unfavorable pulmonary TB treatment outcomes[11]. That score utilized information that was not readily available in SINAN, thus we found it difficult to translate to the nationwide TB program in Brazil. The present study intended to create a score that could be employed in all settings, especially in those with limited resources, which could certainly help guide interventions at the moment of diagnosis, before starting treatment in a large country such as Brazil.

Our risk model had several limitations. First, the study utilized nationwide public data, and several features had missing data and were exposed to a wide range of demographic and regional discrepancies. Second, most co-morbidities and clinical characteristics were self-reported, which may provide potential misclassification bias. Also, the study included only pulmonary TB cases and consequently may not be applied to extrapulmonary or disseminated TB.

Despite the limitations, to the best of our knowledge, this is the first prognostic score model developed in South America using only clinical and epidemiologic data from disease notification forms, obtained before therapy initiation, with relatively accurate prediction. The resulting model is parsimonious and should be utilized by clinicians through a nomogram or web application (https://tbprediction.onrender.com), assisting in TB care and potentially improving the successful completion of ATT of pulmonary TB patients.

## Figures and Tables

**Table 1:** Characteristics of the overall population of the study

Characteristics	Overall Population	Cure	Loss to follow-up	p-value
	*N=243,726*	*N=202,353*	*N=41,373*	
**Age**	42.1(16.3)	43.1 (16.6)	37.1(14.0)	0.000
**Biologic Sex:**				< 0.001
Female	84381 (34.6%)	73380(36.3%)	11001(26.6%)	
Male	159334(65.4%)	128965(63.7%)	30369(73.4%)	
Missing	11(0.00%)	8(0.00%)	3(0.01%)	
**Race:**				< 0.001
White	69388(28.5%)	60333(29.8%)	9055(21.9%)	
Non-white	162314(66.6%)	132193(65.3%)	30121(72.8%)	
Missing	12024(4.93%)	9827(4.86%)	2197(5.31 %)	
**Schooling**				0.000
< 5 years	30685(12.6%)	25757(12.7%)	4928(11.9%)	
[5,9) years	57397(23.5%)	45487(22.5%)	11910(28.8%)	
[9,12) years	74104(30.4%)	62998(31.1%)	11106(26.8%)	
≥ 12 years	13873(5.69%)	12908(6.38%)	965(2.33%)	
Missing	67667(27.8%)	55203(27.3%)	12464(30.1%)	
**Alcohol Use**	44556(18.3%)	32546(16.1%)	12010(29.0%)	0.000
Missing	8439(3.46%)	6656(3.29%)	1783(4.31%)	
**Diabetes:**	24398(10.0%)	22054(10.9%)	2344(5.67%)	< 0.001
Missing	8850(3.63%)	6875(3.40%)	1975(4.77%)	
**Mental Illness**	5961 (2.45%)	4797(2.37%)	1164(2.81 %)	< 0.001
Missing	9259(3.80%)	7242(3.58%)	2017(4.88%)	
**Drug Use**	30290(12.4%)	18445(9.12%)	11845(28.6%)	0.000
Missing	9846(4.04%)	7765(3.84%)	2081(5.03%)	
**HIV infection**	17575(7.21 %)	12124(5.99%)	5451(13.2%)	0.000
Missing	35611(14.6%)	27091 (13.4%)	8520(20.6%)	
**Prior TB**	35279(14.5%)	21858(10.8%)	13421(32.4%)	0.000
**Tobacco Use:**	58705(24.1 %)	44233(21.9%)	14472(35.0%)	0.000
Missing	8704(3.57%)	6842(3.38%)	1862(4.50%)	
**DOT**	92539(38.0%)	83778(41.4%)	8761 (21.2%)	0.000
Missing	48856(20.0%)	37263(18.4%)	11593(28.0%)	

**Table note:** Data represent no. (%), except for age, which is presented as median and interquartile range (IQR).

*Definition of alcohol use:* Past or current any consumption of alcohol.

*Definition of smoking:* Past or current smoking of tobacco.

*Definition of non-white race:* combination of black, mixed, *pardo*, yellow and indigenous.

*Definition of drug use:* Past or current drug use (marijuana, cocaine, heroin, or crack).

*Other comorbidities:* Include cancer, kidney disease, chronic obstructive pulmonary disease, emphysema, allergies, and asthma.

Abbreviations: TB: tuberculosis; PTB: Pulmonary tuberculosis; DOT: Directly Observed Therapy; EPTB: Extrapulmonary tuberculosis.

**Table 2:** Comparison between the models.

Model	Accuracy	Sensitivity	Specificity	PPV	NPV
Logistic Regression	0.67	0.58	0.75	0.70	0.64
Random Forest	0.67	0.62	0.60	0.69	0.66
Light Gradient Boosting	0.67	0.62	0.72	0.69	0.66

Abbreviations: PPV: Positive predictive value; NPV: Negative predictive value

## Data Availability

The datasets generated and/or analyzed during the current study are available in the ***github*** repository, available in the link: https://github.com/rodriguesmsb/TBPrediction
